# A regulatory miRNA–mRNA network is associated with transplantation response in acute kidney injury

**DOI:** 10.1186/s40246-021-00363-y

**Published:** 2021-12-09

**Authors:** Duan Guo, Yu Fan, Ji-Rong Yue, Tao Lin

**Affiliations:** 1 Department of Palliative Medicine, West China School of Public Health and West China fourth Hospital, Sichuan University, Chengdu, 610041 China; 2 Palliative Medicine Research Center, West China-PUMC C.C. Chen Institute of Health, Sichuan University, Chengdu, 610041 China; 3 Department of Urology, National Clinical Research Center for Geriatrics and Organ Transplantation Center, West China Hospital of Sichuan University, No. 37 Guoxue Xiang, Chengdu, 610041 China; 4 Department of Geriatrics and National Clinical Research Center for Geriatrics, West China Hospital, Sichuan University, Chengdu, 610041 China

**Keywords:** Acute kidney injury, Renal transplantation, WGCNA, miRNA–mRNA network

## Abstract

**Background:**

Acute kidney injury (AKI) is a life-threatening complication characterized by rapid decline in renal function, which frequently occurs after transplantation surgery. However, the molecular mechanism underlying the development of post-transplant (post-Tx) AKI still remains unknown. An increasing number of studies have demonstrated that certain microRNAs (miRNAs) exert crucial functions in AKI. The present study sought to elucidate the molecular mechanisms in post-Tx AKI by constructing a regulatory miRNA–mRNA network.

**Results:**

Based on two datasets (GSE53771 and GSE53769), three key modules, which contained 55 mRNAs, 76 mRNAs, and 151 miRNAs, were identified by performing weighted gene co-expression network analysis (WGCNA). The miRDIP v4.1 was applied to predict the interactions of key module mRNAs and miRNAs, and the miRNA–mRNA pairs with confidence of more than 0.2 were selected to construct a regulatory miRNA–mRNA network by Cytoscape. The miRNA–mRNA network consisted of 82 nodes (48 mRNAs and 34 miRNAs) and 125 edges. Two miRNAs (miR-203a-3p and miR-205-5p) and ERBB4 with higher node degrees compared with other nodes might play a central role in post-Tx AKI. Additionally, Gene Ontology (GO) and Kyoto Encyclopedia of Genes and Genomes (KEGG) pathway analysis indicated that this network was mainly involved in kidney-/renal-related functions and PI3K–Akt/HIF-1/Ras/MAPK signaling pathways.

**Conclusion:**

We constructed a regulatory miRNA–mRNA network to provide novel insights into post-Tx AKI development, which might help discover new biomarkers or therapeutic drugs for enhancing the ability for early prediction and intervention and decreasing mortality rate of AKI after transplantation.

**Supplementary Information:**

The online version contains supplementary material available at 10.1186/s40246-021-00363-y.

## Background

As a type of clinical critical illness with rapid loss of renal function and high mortality, acute kidney injury (AKI) commonly occurs in transplant recipients, which might result in transplant failure and death [1]. Timely diagnosis and treatment are crucial in improving prognosis of patients with AKI but are currently impeded by the lack of specific indicators for early prediction, graded evaluation, and monitoring of curative effect. Since AKI is the most common critical illness in multidisciplinary fields, a mounting number of studies about AKI were reported in past decades [2–4]. However, the pathogenesis of AKI is still unclear.

A microRNA (miRNA) is a kind of small non-coding RNA containing approximately 22 nucleotides, which can bind to the 3′-UTR of the target mRNAs at the post-transcriptional level to exert various important physiological and pathophysiological functions in cells [5]. It was reported that miRNAs are capable of regulating a variety of mammalian mRNAs [6], while a single mRNA could be targeted by a large group of miRNAs, demonstrating that the roles of miRNA in gene regulation should be interpreted by complex networks [7]. In recent years, studies for mRNA–miRNA network have increased exponentially, as it is believed to help uncover the molecular mechanism of various diseases, which included neuroblastoma [8], type 2 diabetes [9], and spontaneous intracerebral hemorrhage [10]. Recent studies have found that the changes in the expression of mRNA and miRNA would affect proliferation and apoptosis of renal cells, which are related to the occurrence and development of AKI [11, 12]. Nevertheless, there are few data published on the potential network of mRNA and miRNA in AKI following transplantation.

In the era of precision medicine, high-throughput sequencing data combined with effective bioinformatics analysis can identify potential target genes and mechanisms that contribute to the progress of AKI. The weighted gene co-expression network analysis (WGCNA) is a method widely used to find the core regulators of diseases, as it has the capacity of clustering genes with similar expression patterns into modules (wherein core regulators are commonly found) and analyzing the relationship between modules and specific traits or phenotypes [13]. In a newly published study of cervical intraepithelial neoplasia (CIN), WGCNA was performed to identify six disease-associated modules, from which 31 candidate hub genes for CIN treatment were screened [14]. Bioinformatics analysis not only improves the efficiency of research on biological functions, but also provides reliable information for exploring molecular mechanisms [15, 16]. Based on the large datasets of both mRNA and miRNA expression profiles in the same patient, exploring the regulatory miRNA–mRNA network could help elucidate the molecular mechanisms of the diseases [17, 18].

In this study, the GSE53769 (mRNA) and GSE53771 (miRNA) expression datasets were, respectively, subjected to WGCNA to identify key modules associated with post-Tx AKI. Next, a regulatory miRNA–mRNA network was constructed to clarify the epigenetic mechanisms underlying the progression of post-Tx AKI, thereby providing a possible direction for future clinical research.

## Results

### Identification of key modules related to post-Tx AKI based on GSE53769 dataset

**Fig. 1:**
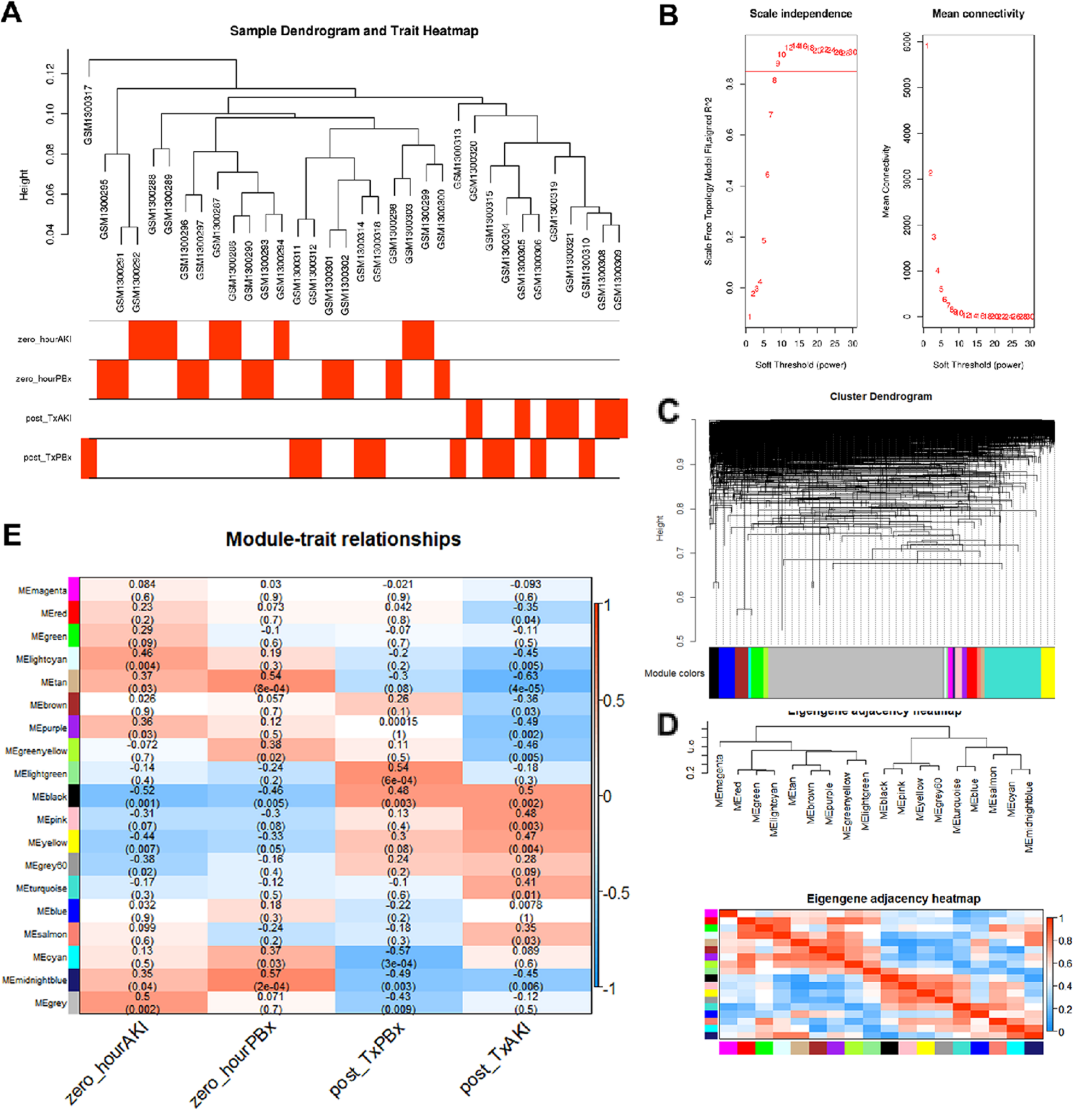
Weighted gene co-expression network analysis (WGCNA) based on GSE53769 dataset. **a** Sample clustering and trait heatmap in GSE53769. **b** Determination of soft-thresholding power (*β*) by analyzing (left) scale-free fit index and (right) mean connectivity. The *β* was set as 9 for constructing a scale-free co-expression network. **c** Dendrogram of consensus module eigengenes. **d** Hierarchical clustering dendrogram and a heatmap of the adjacencies in the eigengene network. **e** Heatmap of the correlation between module eigengenes and different clinical traits

According to Pearson’s correlation and average linkage algorithms, 36 samples were clustered and the sample dendrogram and trait heatmap are depicted in Fig. 1a; we found that GSM1300317 (which belongs to post-Tx PBx group) was clustered alone and might be a potential outlier. Therefore, a t-SNE (t-distributed stochastic neighbor embedding) plot was used to make sure that this sample will not affect the subsequent analyses. As shown in Additional file 2: Figure S2, there was no obvious outlier after the dimension reduction, and therefore, we proceeded with the WGCNA. As shown in Fig. 1b, the soft-threshold power *β* of 9 was selected to guarantee the scale-free character of gene co-expression network (Fig. 1b). The histogram of network connectivity and the corresponding log–log plot are shown in Additional file 3: Figure S3A-B; the *R*^2^ was 0.89, indicating that an approximate scale-free topology was satisfied. Detailed information of soft threshold fit indices including *k*, *R*^2^, and fitted *R*^2^ is provided in Additional file 10: Table S1. Then, through the average linkage hierarchical clustering, genes with similar expression patterns were divided into modules (Fig. 1c). To better distinguish modules with different expression patterns, each module was allocated different colors. As shown in Fig. 1d, a module clustering dendrogram was constructed which generated a total of 18 modules. The gray module contained the genes that cannot be assigned to other 17 modules. A heatmap describing the correlation between clinical traits and modules is shown in Fig. 1e. Among these modules, the black module displayed the highest positive correlation with post-Tx AKI (*P* = 0.002, *R* = 0.5), while the tan module displayed the strongest negative correlation with post-Tx AKI (*P* = 4e − 05, *R* =  − 0.63). Thus, these two modules were selected as key modules. Assignments of mRNAs in black and tan modules are provided in Additional file 11: Table S2.

### Gene–gene network and functional enrichment analysis in the black and tan modules

**Fig. 2:**
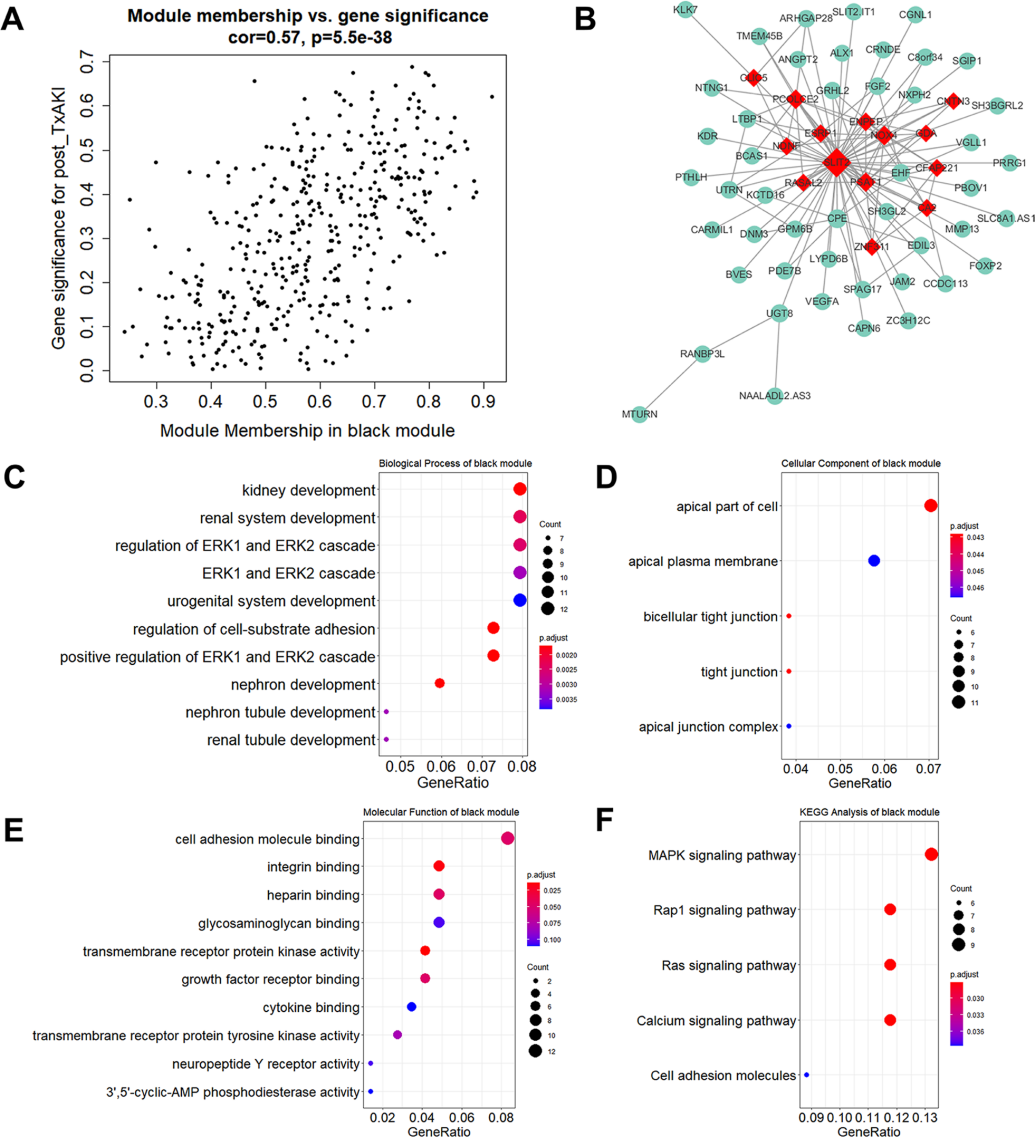
Analysis of the black module positively associated with post-Tx AKI. **a** Scatter plot of mRNAs in the black module. **b** The connectional network of the black module mRNAs and 12 hub mRNAs was identified as red. Functional annotation of the black module mRNAs, which included the analysis of **c** GO-BP, **d** GO-CC, **e** GO-MF, and **f** KEGG pathway. GO, Gene Ontology; BP, biological process; CC, cellular component; MF, molecular function; and KEGG, Kyoto Encyclopedia of Genes and Genomes.

As shown in Fig. 2a, the correlation coefficient of module membership (MM) vs. gene significance (GS) in the black module was 0.57 with *P* = 5.5e − 38. A total of 14 hub genes were identified in the black module, which included CLIC5, PCOLCE2, NDNF, ESRP1, ENPEP, RASAL2, SLIT2, PSAT1, NOX4, GDA, CNTN3, CFAPP221, CA2, and ZNF311 (Fig. 2b). Then, the GO and KEGG pathway enrichment analysis was performed on the genes in the black module. As shown in Fig. 2c, we found the most enriched GO terms in the category of biological process (GO-BP) were kidney development, renal system development, and regulation of ERK1/2 cascade. The most enriched GO terms in the other categories (GO-CC and GO-MF) were the apical part of cell and cell adhesion molecule binding (Fig. 2d, e). For KEGG pathway analysis, these genes were mainly enriched in MAPK signaling and Raq1 signaling pathways (Fig. 2f).

**Fig. 3:**
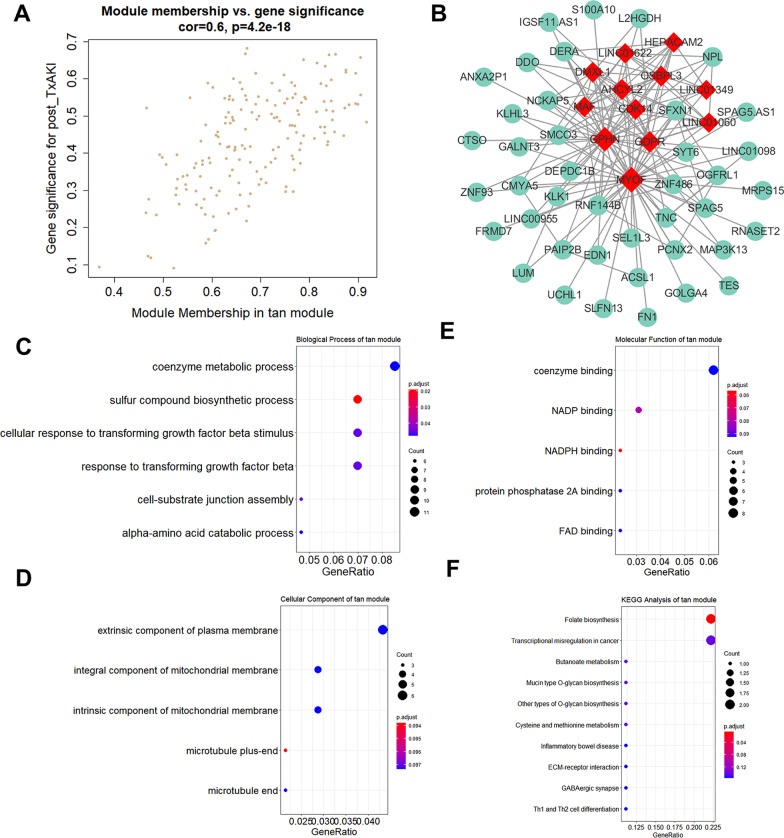
Analysis of the tan module negatively associated with post-Tx AKI. **a** Scatter plot of mRNAs in the tan module. **b** The connectional network of the tan module mRNAs and 12 hub mRNAs was identified as red. Functional annotation of the tan module mRNAs, which contained analysis of **c** GO-BP, **d** GO-CC, **e** GO-MF, and **f** KEGG pathway. GO, Gene Ontology; BP, biological process; CC, cellular component; MF, molecular function and KEGG, Kyoto Encyclopedia of Genes and Genomes

The genes of the tan module underwent the same analysis. The scatter plots of MM versus GS in the tan module (cor = 0.6, *P* = 4.2e − 18) are shown in Fig. 3a. The gene–gene network centered on hub genes in this module is depicted in Fig. 3b. As we can see, the tan module contained 12 hub genes including DMXL1, MAF, GPHN, MYOF, CDK14, and QDPR. The functional annotation of genes in the tan module is depicted in Fig. 3c–f, indicating that the black module genes were primarily enriched in functions of coenzyme metabolic process, extrinsic component of plasma membrane, and coenzyme binding, as well as pathways of folate (FA) biosynthesis. Detailed information of differentially expressed genes (defined by log2 fold change > 0.1 and *p*-value < 0.05 when comparing their expression in post-Tx AKI group to that in zero-hour AKI group) in black module and tan module is provided in Additional file 4: Figure S4 and Additional file 5: Figure S5, respectively.

### Identification of key modules related to post-Tx AKI based on GSE53771 dataset

**Fig. 4:**
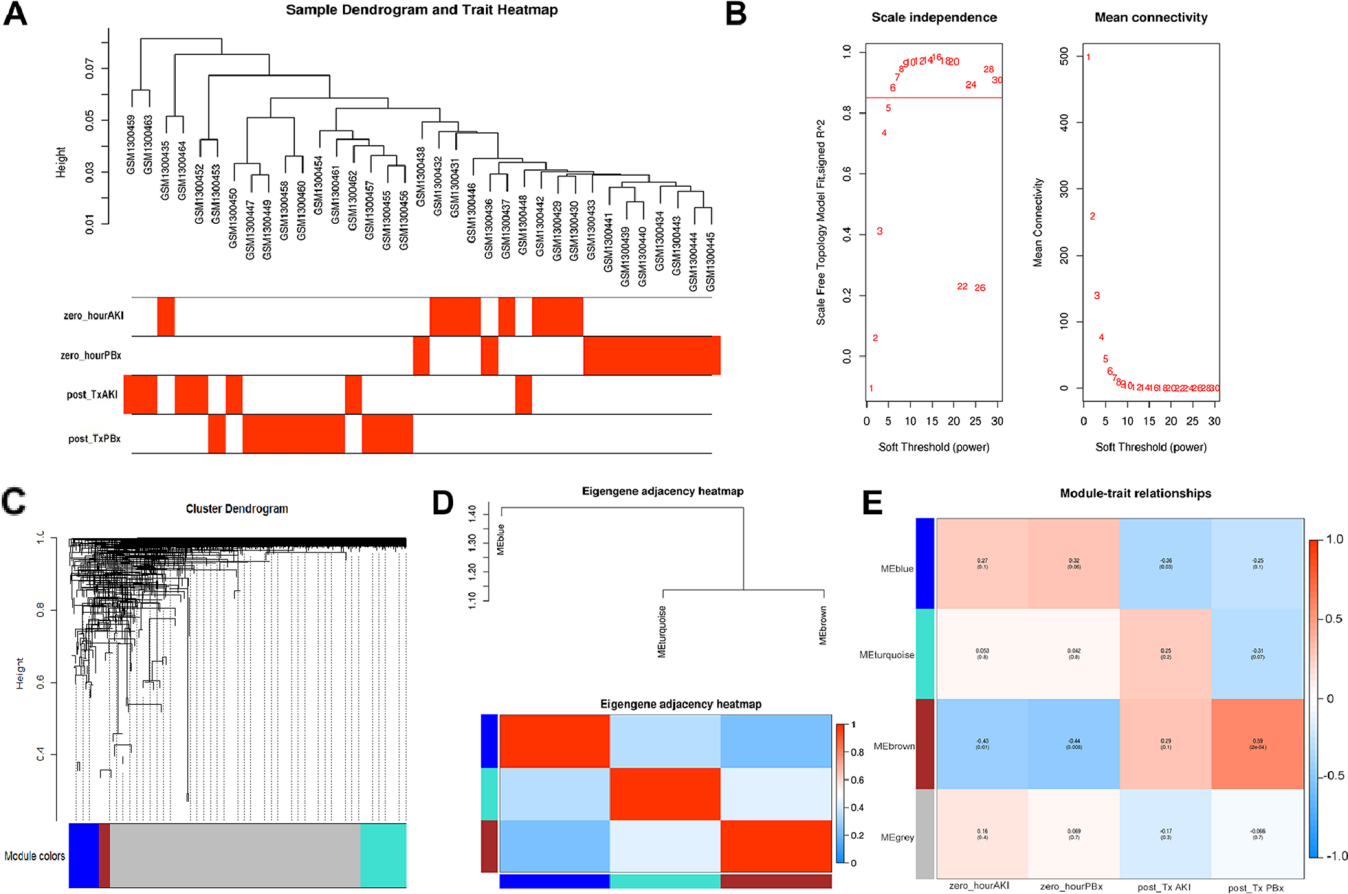
Weighted gene co-expression network analysis (WGCNA) based on GSE53771 dataset. **a** Sample clustering and trait heatmap in GSE53771. **b** Determination of soft-thresholding power (*β*) by analyzing (left) scale-free fit index and (right) mean connectivity. The *β* was set as 6 for constructing a scale-free co-expression network. **c** Dendrogram of consensus module eigengenes. **d** Hierarchical clustering dendrogram and a heatmap of the adjacencies in the eigengene network. **e** Heatmap of the correlation between module eigengenes and different clinical traits

In order to explore the roles of miRNA in post-Tx AKI, we also conducted WGCNA for miRNAs based on GSE53771 dataset. The construction steps of co-expression network for miRNAs were similar to those for mRNAs. The sample dendrogram and trait heatmap are shown in Fig. 4a. To ensure a scale-free network, the soft-threshold power *β* was set as 6 (Fig. 4b). The histogram of network connectivity and the corresponding log–log plot are shown in Additional file 3: Figure S3C-D; the *R*^2^ was 0.98, indicating that an approximate scale-free topology was satisfied. Detailed information of soft-threshold fit indices including *k*, *R*^2^, and fitted *R*^2^ is provided in Additional file 10: Table S1. A total of three miRNA modules were identified, which were independent from each other (Fig. 4c, d). Then, the correlation of miRNA modules with clinical traits was analyzed, and the result showed that the blue miRNA module was the only module significantly correlated with post-Tx AKI (*P* = 0.003, *R* =  − 0.36) (Fig. 4e). Therefore, the blue miRNA module which consisted of 76 miRNAs was chosen as the key miRNA module for subsequent analysis. Detailed information of these miRNAs is provided in Additional file 11: Table S2.

### MiRNA–miRNA network and functional enrichment analysis in the blue miRNA module

**Fig. 5:**
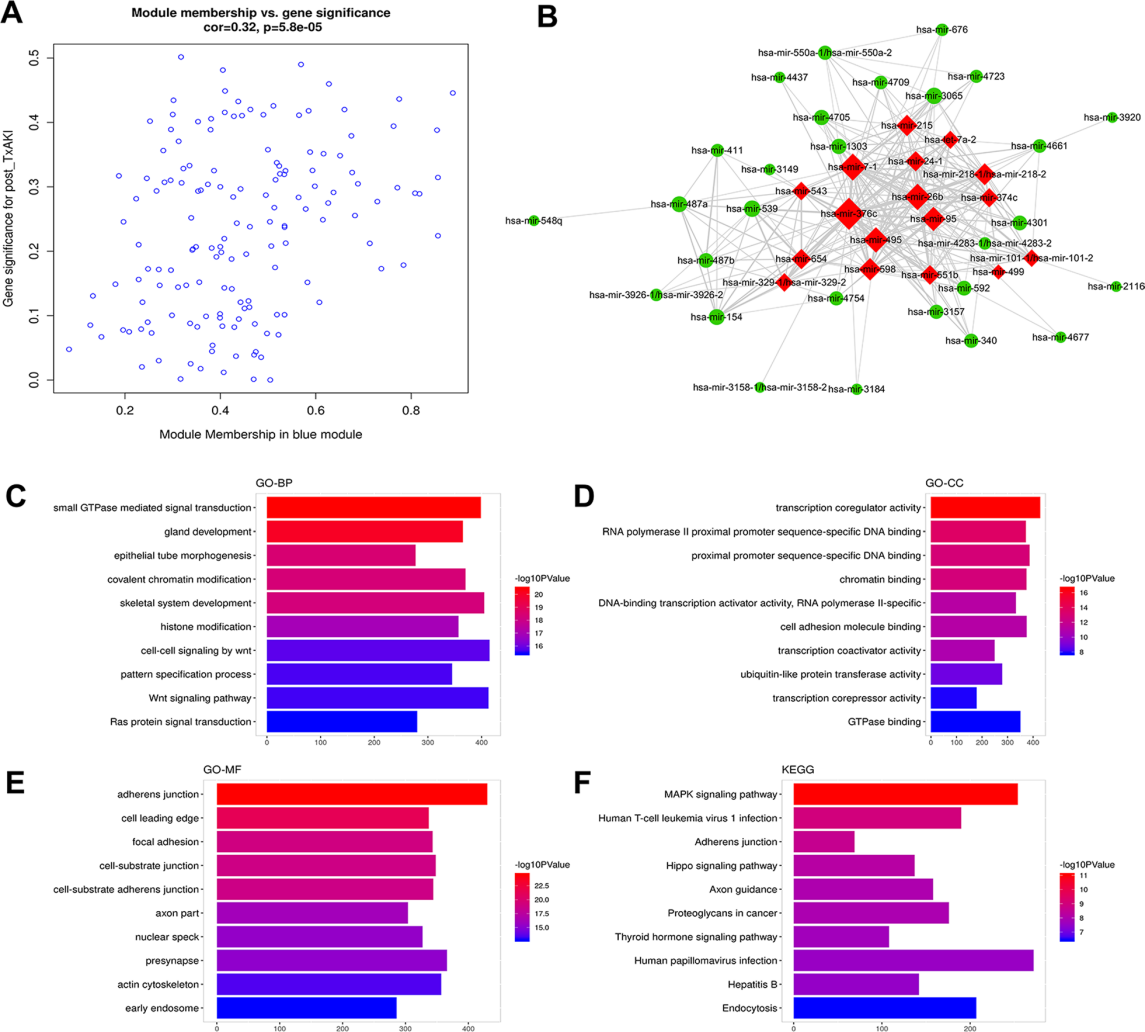
Analysis of the blue miRNA module which negatively associated with post-Tx AKI. **a** Scatter plot of miRNAs in the blue miRNA module. **b** The connectional network of the blue module miRNAs and 17 hub miRNAs was identified as red. Functional annotation of the targets of blue module miRNAs, which contained analysis of **c** GO-BP, **d** GO-CC, **e** GO-MF, and **f** KEGG pathway. The targets of blue module miRNAs were predicted by “miRNAtap” and “multiMiR” R packages. GO, Gene Ontology; BP, biological process; CC, cellular component; MF, molecular function; and KEGG, Kyoto Encyclopedia of Genes and Genomes

As shown in Fig. 5a, the correlation coefficient of MM versus GS in the blue miRNA module was 0.32 with *P* = 5.8e − 5. The interaction network of module miRNAs showed that 17 hub miRNAs were identified in the blue miRNA module (Fig. 5b). To further explore the biological roles of miRNAs in this module, the target genes of these miRNAs were used to perform functional enrichment analysis. Results of function annotation are shown in Fig. 5c–e, suggesting that the miRNAs of blue miRNA module were significantly associated with the GO terms of small GTPase mediated transduction, gland development, transcription coregulator activity, and adherens junction, as well as the KEGG items of MAPK signaling pathway and human T-cell leukemia virus 1 infections. Detailed information of differentially expressed miRNAs (defined by log2 fold change > 0.1 and *p*-value < 0.05 when comparing their expression in post-Tx AKI group to that in zero-hour AKI group) in blue module is provided in Additional file 6: Figure S6.

### Construction of regulatory miRNA–mRNA network in post-Tx AKI

**Fig. 6:**
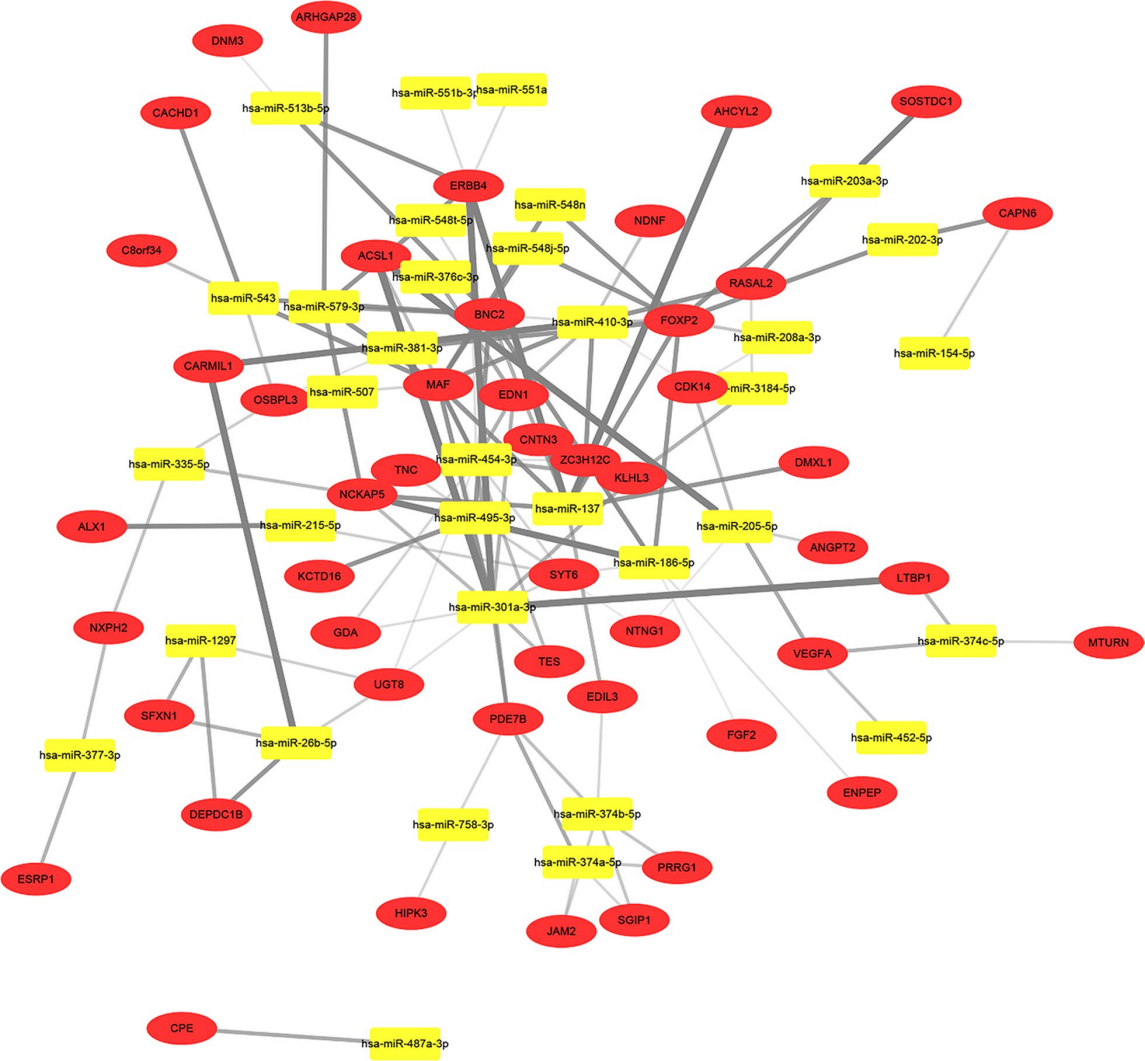
The regulatory miRNA–mRNA network associated with post-Tx AKI (interaction confidence ≥ 0.2). There are 48 mRNAs nodes, 34 miRNAs nodes, and 125 edges in the network. Red ellipses represent mRNAs; yellow round rectangles represent miRNAs. The thickness of edge indicates the strength of correlation between mRNA and miRNA

**Fig. 7:**
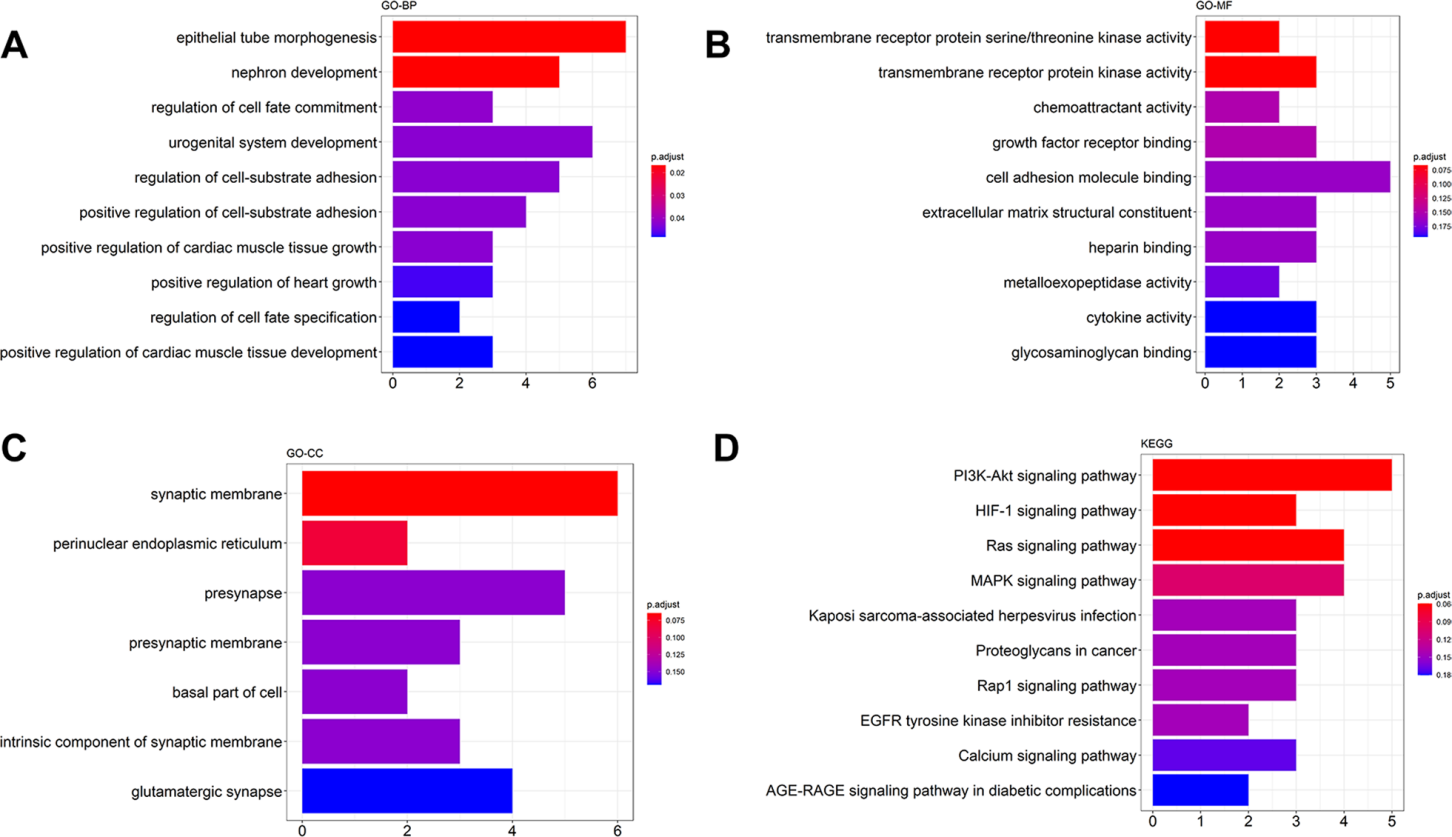
Functional enrichment analysis of the regulatory miRNA–mRNA network. **a** GO-BP, **b** GO-CC, **c** GO-MF, and **d** KEGG pathway. GO, Gene Ontology; BP, biological process; CC, cellular component; MF, molecular function; and KEGG, Kyoto Encyclopedia of Genes and Genomes

The genes and miRNAs that, respectively, formed the key modules and key miRNA module were used to generate the regulatory miRNA–mRNA network. A total of 1048 predicted miRNA–mRNA pairs in high confidence class were obtained from miRDIP v4.1 and used to construct a network by Cytoscape (Additional file 7: Figure S7). The functional annotation of mRNAs in this network is depicted in Additional file 8: Figure S8, which indicates that these mRNAs were mainly involved in cell–substrate adhesion, urogenital system development, heparin binding, collagen binding, AGE–RAGE signaling pathway in diabetic complications, as well as PI3K–Akt signaling pathway. Afterward, to obtain the network that may exert key roles in the pathogenesis of post-Tx AKI, we selected the miRNA–mRNA pairs with confidence of more than 0.2 to generate a regulatory miRNA–mRNA network (Fig. 6). The regulatory miRNA–mRNA network was comprised of 82 nodes (48 mRNAs and 34 miRNAs) and 125 edges. After analyzing the network, we found miR-203a-3p, miR-205-5p, and ERBB4 with higher node degrees compared with other nodes, and the quantification details are provided in Additional file 12: Table S3. Then, functional analysis revealed that the total of 48 mRNAs were mainly enriched in GO terms of epithelial tube morphogenesis, nephron development, urogenital system development, and transmembrane receptor protein kinase (Fig. 7a–c). There were no significantly enriched KEGG pathways since their p-values were greater than 0.05 (Fig. 7d). For cross-validation, we compared our current results retrieved from miRDIP database to that from miRNet database. In total, 75,699 miRNA–mRNA pairs were identified by miRNet, and there was an intersection of 117 miRNA–mRNA pairs between the results of miRNet database and miRDIP database; the corresponding regulatory network is visualized in Additional file 9: Figure S9B. The enrichment analyses of the 117 miRNA–mRNA pairs showed that they were predominantly involved in negative regulation of cellular component organization, response to organic substance in GO-BP category; eukaryotic translation initiation factor 4F complex, and nuclear body in GO-CC category; SNAP receptor activity, protein complex binding, protein tyrosine phosphatase activity under GO-MF category, as well as KEGG pathways associated with renal cell carcinoma signaling, prolactin signaling and NFR2-mediated oxidative stress response. Since the above functional enrichment results were based on miRNA–mRNA pairs predicted by both miRNet and miRDIP databases, they might be more representative for the epigenetic mechanisms underlying the post-Tx AKI.

## Discussion

Post-Tx AKI is a common complication after transplantation surgery, which is characterized by rapid decline in renal function and high mortality [19]. Grasping the best time for early diagnosis and intervention of post-Tx AKI is challenging due to the lack of specific indicators for early prediction, grading assessment, and monitoring of efficacy. To overcome such issues, exploring the pathomechanisms and potential biomarkers of post-Tx AKI is crucial. In 2014, Wilflingseder et al. conducted miRNA and mRNA microarray analysis based on renal transplant biopsy specimen with AKI and further identified an AKI-specific molecular signature using differential gene expression analysis (DEA) [20]. However, DEA can easily exclude some important genes whose expression level changes little, but play a crucial role in the diseases. Besides, it is difficult to confirm whether the differentially expressed mRNAs and miRNA in the post-Tx biopsy between control and AKI were related to post-Tx AKI due to the fact that Tx also could lead to the abnormal expression of some genes. Increasing studies demonstrated that the initiation and progression of all diseases could not be regulated by a few genes, rather a network of multiple RNAs [21, 22]. Thus, constructing an RNA regulatory network might be a promising strategy for understanding disease development and establishing new therapy [23]. As a robust bioinformatics approach, WGCNA has the capacity to enhance simple correlation networks by quantifying the correlations between individual pairs of genes, as well as the extent to which these genes share the same neighbors [24]. WGCNA includes not only differentially expressed genes, but also genes that are not significantly differentially expressed, but still a key mediator of certain clinical traits. In recent years, WGCNA has been applied in a diverse range of human disease researches to screen biomarkers and clarify molecular mechanisms underlying disease development [25, 26].

In the current study, based on the mRNA and miRNA microarray datasets, three modules which were significantly correlated with AKI following transplantation were identified by using WGCNA. The tan module containing 55 mRNAs showed a significantly negative correlation with post-Tx AKI. Multiple studies reported that high concentration of FA could produce acute tubular necrosis as the generation of FA crystals in renal tubules, leading to renal failure [27, 28]. The mRNAs in the tan module were mainly involved in the pathway of FA biosynthesis, suggesting that this pathway accounts for the development of post-Tx AKI. Next, as the module most positively related to post-Tx AKI, the black module was comprised of 80 mRNAs. Notably, the most enriched items of the black module mRNAs in GO analysis were mainly related to the renal functions such as kidney development and nephron development, confirming the high correlation of this module with post-Tx AKI. A previous study indicated that extracellular signal-regulated kinase (ERK) cascade plays a fundamental role in the activation of compensatory repair mechanisms during kidney injury [29]. Our study showed that the black module mRNAs were also significantly enriched in the functions of ERK1/2 cascade. Besides, results of KEGG pathway analysis indicated that these mRNAs were closely related to MAPK signaling pathway and Ras signaling pathway. It is largely documented that the MAPK pathway exerts a key role in AKI by regulating renal inflammation, tubular injury, and cell death [30–32]. It is universally accepted that the MAPK/ERK pathway is downstream signal molecule in the Ras signaling pathway [33]. The above results revealed that the abnormal expression of some genes results in AKI by regulating FA biosynthesis, MAPK signaling pathway, and Ras signaling pathway to affect renal development after kidney transplantation.

Increasing experimental evidence has confirmed that certain miRNAs have critical roles in the detection, progression, and intervention of AKI [34]. Amrouche et al. demonstrated that miR-146a has an important role in the renal tubular response, of which upregulation could limit the development of AKI [35]. A previous study proved that urinary miR-21 could be used as a biomarker for predicting AKI development after cardiac surgery [36]. As the only miRNA module significantly associated with post-Tx AKI in this study, the blue miRNA module containing 151 miRNAs was negatively correlated with post-Tx AKI. It has been widely acknowledged that miRNAs can regulate the expression of their downstream target genes to exert biological functions. Accordingly, we predicted the targets of these miRNAs using “miRNAtap” and “multiMiR” to perform functional annotation. It should be noted that the targets of key module miRNAs were also mainly enriched in MAPK signaling pathway, which further confirmed the crucial role of MAPK pathway in the process of post-Tx AKI.

Moreover, it is necessary to identify regulatory miRNA–mRNA network that is potentially involved in the pathogenesis of post-Tx AKI as neither genes nor miRNAs can independently regulate the development of post-Tx AKI. The majority of the previous studies have solely focused on miRNAs or genes to clarify the mechanism of AKI. By using bioinformatics tools, we finally established a regulatory miRNA–mRNA network of post-Tx AKI, which was comprised of 48 mRNAs and 34 miRNAs. Among these 82 nodes, miR-203a-3p, miR-205-5p, and ERBB4 showed high degrees and might be central nodes in the network. The effects of miR-205-5p in renal diseases have been investigated previously. Schena et al. reported that the expression level of miR-205-5p was significantly and positively correlated with the severity of renal cancer and hypertensive nephrosclerosis [37]. An experimental study conducted by Sessa and his colleagues proposed that miR-205-5p could be a molecular biomarker of renal damage [38]. Few studies have investigated the roles of miR-203a-3p and ERBB4 in AKI development. It is documented that ERBB4 could alleviate the oxidative insults of aged mesenchymal stem cells by reducing reactive oxygen species (ROS) levels [39]. It is well known that all transplanted organs will undergo a certain degree of ischemia–reperfusion injury mediated by high level of ROS after transplantation and potentially develop into AKI. We speculated that ERBB4 also exerts the function of regulating ROS levels in the development of post-Tx AKI. The GO enrichment analysis of the mRNAs in this network showed that these mRNAs were enriched in several functions relevant to the renal development. Furthermore, KEGG enrichment analysis indicated that this network was mainly involved in a range of pathways that have been well studied, such as PI3K–Akt signaling pathway, HIF-1 signaling pathway, Ras signaling pathway, and MAPK signaling pathway. Most of these pathways have been proven of crucial roles in AKI [30, 40, 41]. These results proved that our analysis was properly conducted.

Taken together, our study, for the first time, utilized WGCNA combined with miRDIP v4.1 analysis to comprehensively identify the most likely interactions and construct a regulatory miRNA–mRNA network associated with transplantation response in AKI, which provided a preliminary framework and some novel insight for elucidating molecular mechanism of the development of post-Tx AKI. Nevertheless, some limitations of this study should be mentioned. First, only eight post-Tx AKI biopsy samples were enrolled in the present study, which were not sufficient to draw entirely credible conclusions. Second, the regulatory miRNA–mRNA network requires further studies in clinical and molecular biology experiments for validation. Since it is hard to find qualified data, other types of RNAs, such as long non-coding RNAs (lncRNAs) and circular RNAs (circRNAs), were not included, which might be a disadvantage in the comprehensive clarification of the mechanism underlying post-Tx AKI development.

## Conclusions

We first successfully constructed a regulatory miRNA–mRNA network associated with post-Tx AKI by using bioinformatics analysis. The results indicated that two miRNAs (miR-203a-3p and miR-205-5p) and ERBB4 might play a central role in post-Tx AKI, and the biological functions of the regulatory miRNA–mRNA network were enriched in kidney-/renal-related functions and PI3K–Akt/HIF-1/Ras/MAPK signaling pathways. This study provides a comprehensive perspective of regulatory networks to increase the understanding of the molecular mechanism in post-Tx AKI. We hope that the current study will be beneficial for discovering new biomarkers or therapeutic drugs for enhancing the ability for early prediction and intervention and decreasing mortality rate of AKI after transplantation.

## Methods

### Study design and data collection

The overall design of this study is shown in Additional file 1: Figure S1. All eligible microarray data were downloaded from the Gene Expression Omnibus (GEO) database (http://www.ncbi.nlm.nih.gov/geo/). GSE53769 dataset is an mRNA expression dataset which was performed using Affymetrix GeneChip® Human Gene 2.0 ST Array; GSE53771 dataset is a miRNA expression dataset which was analyzed by Affymetrix GeneChip® miRNA 3.0 Array. These two datasets both consisted of 18 zero-hour and 18 post-transplant (Tx) biopsy samples from 18 kidney allograft recipients (eight with acute tubular necrosis without rejection defined as AKI and ten protocol biopsies without pathology (PBx) defined as controls) and were submitted by Wilflingseder and co-workers [20]. These samples were divided into four groups, namely zero-hour AKI, zero-hour PBx, post-Tx AKI, and post-Tx PBx. The two datasets were, respectively, used to construct the co-expression network, whereby key mRNA/miRNA modules associated with post-Tx AKI could be identified, allowing the construction of a miRNA–mRNA regulatory network driving the progression of post-Tx AKI.

### Construction of the co-expression networks

WGCNA is a widely used method to construct co-expression networks that allow the discovery of gene modules, where coordinated expression patterns of the intra-module genes could be identified and related to external clinical phenotypes. In this way, the search for core disease regulators could be narrowed down and confined to the clinically significant modules [13]. Given the foregoing, WGCNA has greatly improved the efficacy of data mining; therefore, in this study, the R package “WGCNA” was utilized to construct co-expression networks based on the expression profiles of mRNAs and miRNAs, respectively. An optimal soft threshold power β, the minimum power parameter that satisfied the scale-free topology (as manifested by scale-free topology fit index > 0.85), was first determined. Next, a scale-free co-expression network was constructed based on the adjacency matrix. The adjacency matrix was obtained using the formula: ${\text{Adjacency}}_{k,j} = \left| {{\text{cor}}\left( {k,j} \right)} \right|^{\beta }$ , where *k* and *j* correspond to two arbitrary genes and the *β* is used to emphasize the strong similarity between *k* and *j*, which ensured that gene pairs with low similarity will be omitted during module assignment. Then, the adjacency matrix was converted into a topological overlap matrix (TOM). By using a TOM-based dissimilarity measure, the gene tree dendrogram was generated by average linkage hierarchical clustering, and genes with similar expression pattern were clustered into different modules (the minimum module size was set to 30).

### Identification of the significant correlation modules

Module eigengenes (MEs), which summarize gene expression pattern as a single characteristic expression profile within a given module, were used to evaluate the potential correlation of genes with different traits for determining the significance of each module. Gene significance (GS) represented the correlation between genes and different clinical traits, and the average GS of all genes in a module was defined as module significance (MS), expressed as: $\mathrm{MS}=\frac{1}{n}{\sum }_{i=1}^{n}{\mathrm{GS}}_{i}$ (*n* = number of genes within a module). After calculating the Pearson correlation between MEs and clinical traits, the modules with the highest positive or lowest negative *R* (correlation coefficient) with post-Tx AKI with correlation *p*-values cutoff of 0.05 were defined as key modules. We followed the standard workflow recommended by the authors of WGCNA [13], and *p*-value correction for multiple testing was not performed since the Pearson coefficient *R* and correlation *p*-value are sufficient for significant module selection [13]. The intensity of the color in the heatmap indicated the strength of correlation. To better study a key module, the correlation of module genes was analyzed and the gene–gene interaction network was visualized by the network analyzer Cytoscape v3.7.2 [42]. In this network, the genes with a high degree, which contained highly interconnected nodes in the module, were considered as hub genes. These hub genes were detected by performing the analysis with the MCODE plugin in Cytoscape.

### Regulatory miRNA–mRNA network construction

By using miRDIP v4.1 online tool (http://ophid.utoronto.ca/mirDIP/) [43], the interactions between the module genes and miRNAs were obtained. Then, the miRNA–mRNA pairs with a high confidence of prediction were chosen for the construction of a miRNA–mRNA regulatory network by using Cytoscape v3.7.2. To cross-validate our results, the miRNA–mRNA interactions were also retrieved from miRNet (https://www.mirnet.ca/) database.

### Functional enrichment analysis

To further understand the potential functions of the identified genes, the enrichment analysis of Gene Ontology (GO) and Kyoto Encyclopedia of Genes and Genomes (KEGG) pathway analysis were performed by using R package “clusterProfiler” [44]; in some cases (Additional file 4: S4A, Additional file 5: S5A, Additional file 6: S6A and Additional file 9: S9C), the function TCGAanalyze_EAcomplete in the TCGAbiolinks library was run. In this study, the results of GO terms and KEGG pathways with the Benjamini–Hochberg (BH) adjusted *P*-values of < 0.05 were considered to be significantly enriched.

## Supplementary Information

**Additional file 1: Figure S1** The overall design of this study.

**Additional file 2: Figure S2** t-SNE plot of GSE53769 dataset.

**Additional file 3: Figure S3**. Confirmation of scale-free characteristics. (**A**) Histogram of connection frequency in GSE53769. (**B**) Log–log plot of whole-network connectivity distribution in GSE53769. (**C**) Histogram of connection frequency in GSE53771. (**D**) Log–log plot of whole-network connectivity distribution in GSE53771.

**Additional file 4: Figure S4**. Detailed information of differentially expressed genes in the black module. (**A**) Significantly enriched GO terms/KEGG pathways of differentially expressed black module genes. (**B**) Heatmap demonstrating the expression profile of black module genes. (**C**) Volcano plot showing the up-/down-regulated black module genes.

**Additional file 5: Figure S5**. Detailed information of differentially expressed genes in the tan module. (**A**) Significantly enriched GO terms/KEGG pathways of differentially expressed tan module genes. (**B**) Heatmap demonstrating the expression profile of tan module genes. (**C**) Volcano plot showing the up- or down-regulated tan module genes.

**Additional file 6: Figure S6**. Detailed information of differentially expressed miRNAs in the blue module. (**A**) Significantly enriched GO terms/KEGG pathways of the target genes of differentially expressed blue module miRNAs. (**B**) Heatmap of demonstrating the expression profile of blue module miRNAs. (**C**) Volcano plot showing the up-/down-regulated blue module miRNAs.

**Additional file 7: Figure S7**. The preliminary miRNA–mRNA network constructed by interactions of module miRNAs and module mRNAs before setting threshold of confidence.

**Additional file 8: Figure S8**. Functional enrichment analysis of the preliminary miRNA–mRNA network. (**A**) GO-BP, (**B**) GO-CC, (**C**) GO-MF, and (**D**) KEGG pathway.

**Additional file 9: Figure S9**. Cross-validation of the miRNA–mRNA regulatory network. (**A**) The total number of the identified miRNA–mRNA pairs was 75699 in miRNet database and 4154 in miRDIP database, respectively. A 117 miRNA–mRNA pairs intersection was found and used to construct the (**B**) miRNA–mRNA regulatory network. The results of the corresponding function enrichment analyses are shown in (**C**).

**Additional file 10: Table S1**. Soft-threshold fit indices of two datasets.

**Additional file 11: Table S2**. The list of miRNA and mRNA in the black, tan, and blue modules.

**Additional file 12: Table S3**. The quantification details of miRNA–mRNA network.

## Data Availability

The datasets GSE53769 (https://www.ncbi.nlm.nih.gov/geo/query/acc.cgi?acc=GSE53769) and GSE53771 (https://www.ncbi.nlm.nih.gov/geo/query/acc.cgi?acc=GSE53771) analyzed during the current study are available in the Gene Expression Omnibus (GEO) datasets at the National Center for Biotechnology Information (http://www.ncbi.nlm.nih.gov/geo/).

## Abbreviations

Tx: Transplant
PBx: Protocol biopsies without pathology
AKI: Acute kidney injury
WGCNA: Weighted gene co-expression network analysis
CIN: Cervical intraepithelial neoplasia
ROS: Reactive oxygen species
miRNA: MicroRNA
GEO: Gene expression omnibus
TOM: Topological overlap matrix
MEs: Module eigengenes
GS: Gene significance
MS: Module significance
GO: Gene Ontology
KEGG: Kyoto Encyclopedia of Genes and Genomes
