# Supplementary figures and images for: A regulatory miRNA–mRNA network is associated with transplantation response in acute kidney injury
Source: Hum Genomics. 2021 Dec 9;15:69. doi: 10.1186/s40246-021-00363-y (PMC8656037; doi:10.1186/s40246-021-00363-y)

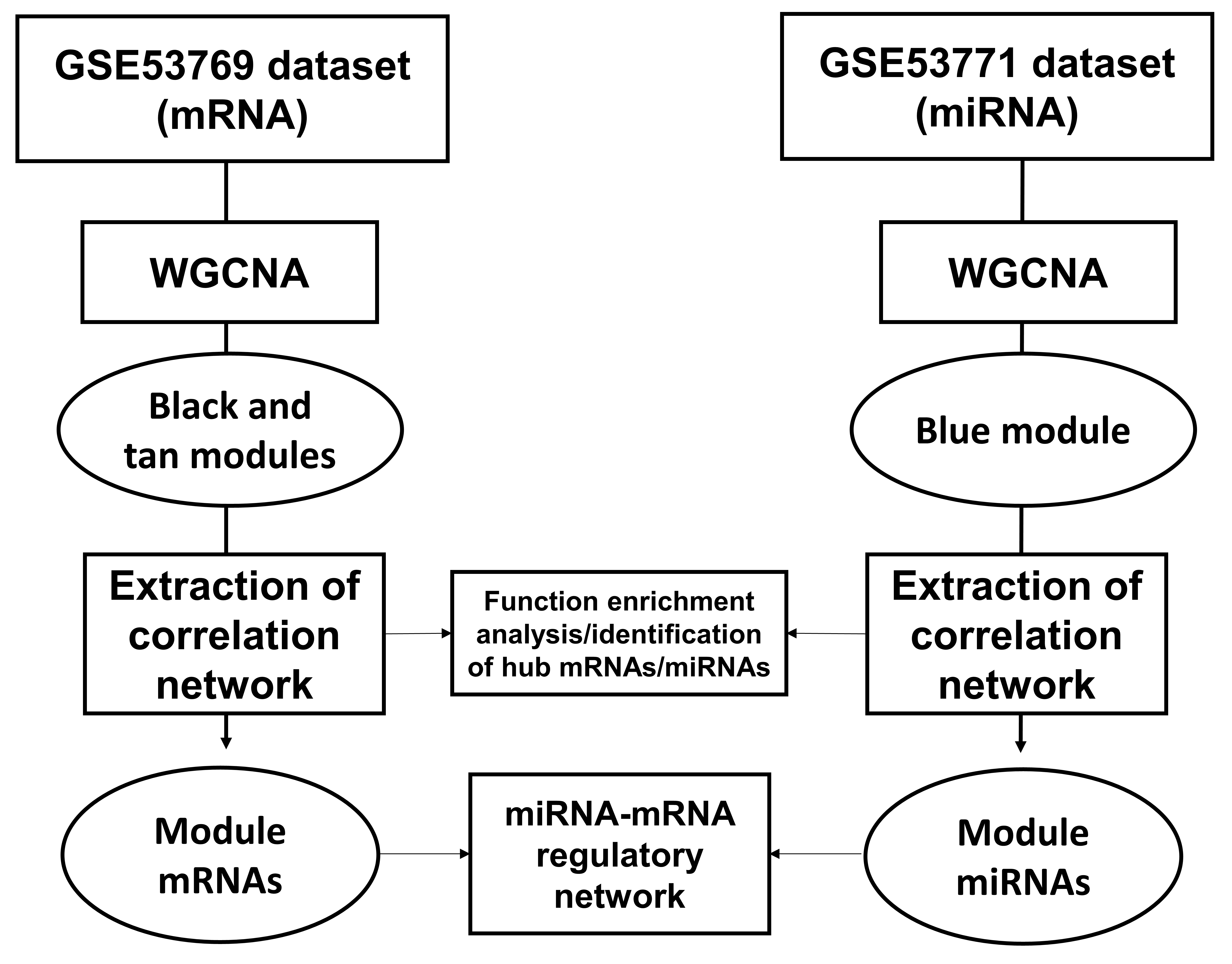

Supplement: Supplementary file 1 — Additional file 1: Figure S1 The overall design of this study. [file 40246_2021_363_MOESM1_ESM.tif]

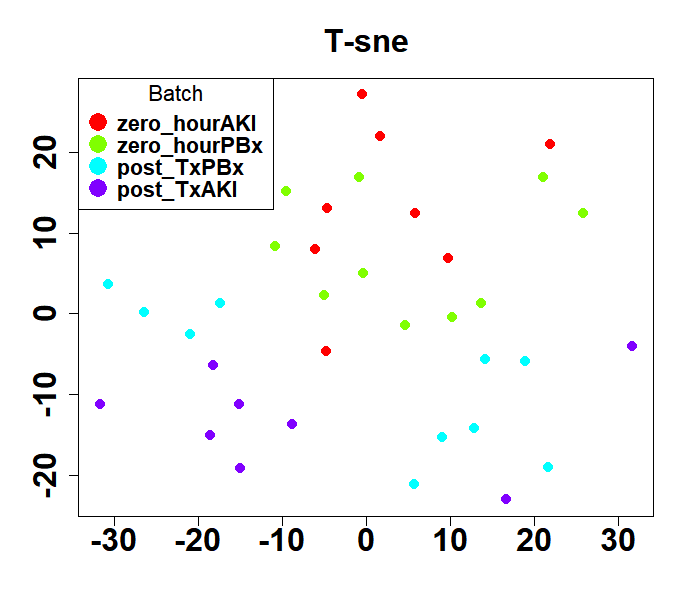

Supplement: Supplementary file 2 — Additional file 2: Figure S2 t-SNE plot of GSE53769 dataset. [file 40246_2021_363_MOESM2_ESM.tif]

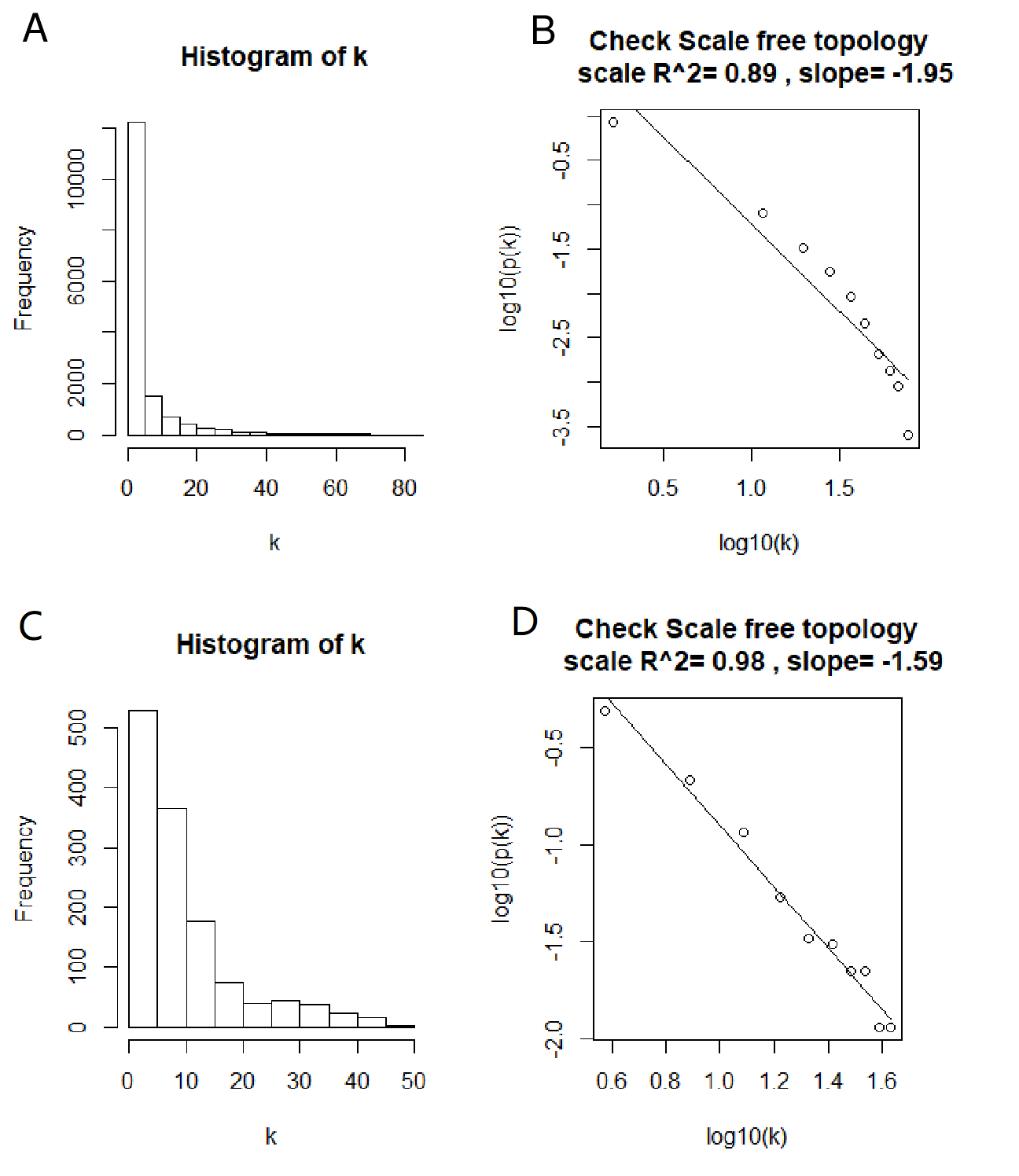

Supplement: Supplementary file 3 — Additional file 3: Figure S3. Confirmation of scale-free characteristics. (A) Histogram of connection frequency in GSE53769. (B) Log–log plot of whole-network connectivity distribution in GSE53769. (C) Histogram of connection frequency in GSE53771. (D) Log–log plot of whole-network connectivity distribution in GSE53771. [file 40246_2021_363_MOESM3_ESM.tif]

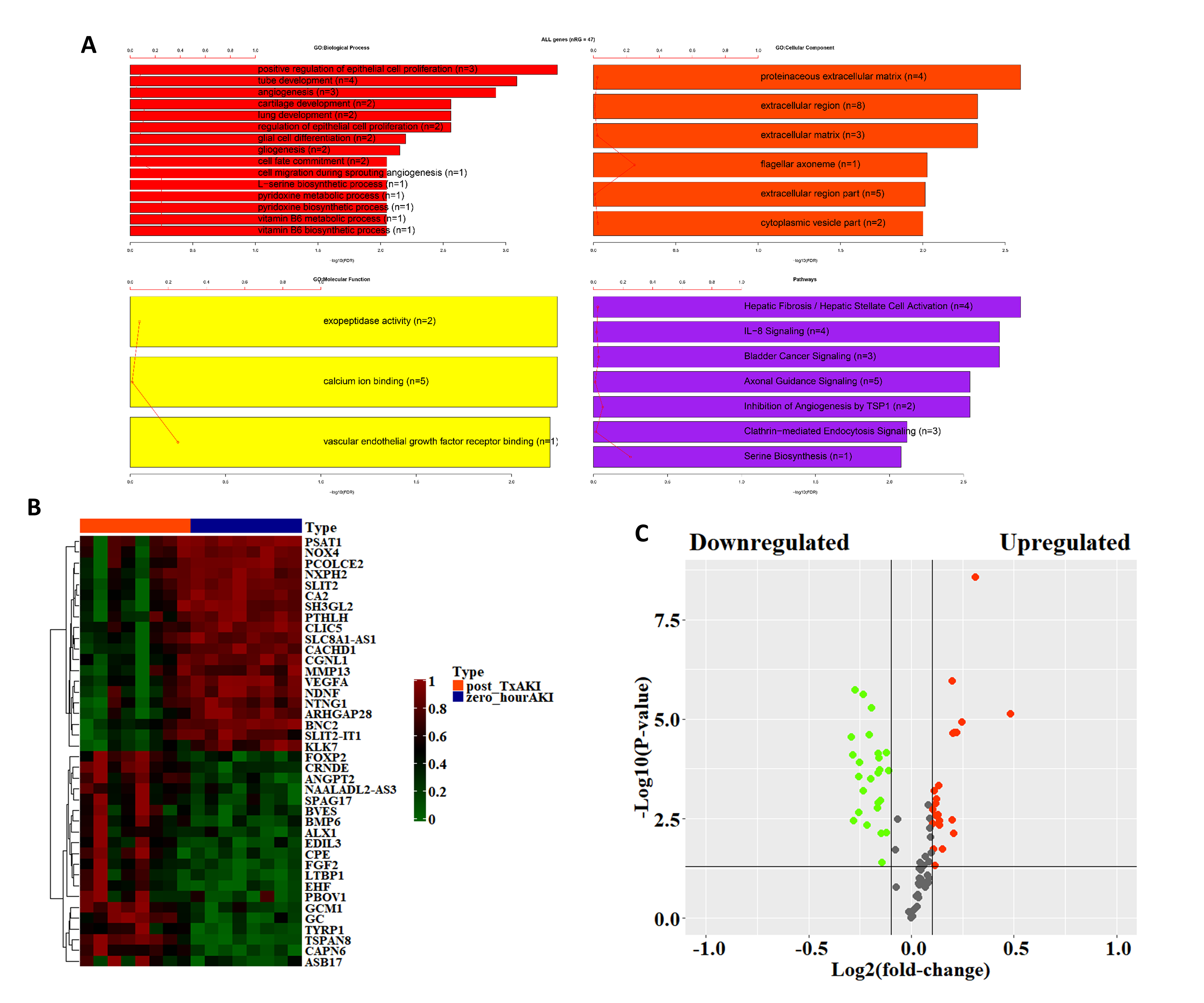

Supplement: Supplementary file 4 — Additional file 4: Figure S4. Detailed information of differentially expressed genes in the black module. (A) Significantly enriched GO terms/KEGG pathways of differentially expressed black module genes. (B) Heatmap demonstrating the expression profile of black module genes. (C) Volcano plot showing the up-/down-regulated black module genes. [file 40246_2021_363_MOESM4_ESM.tif]

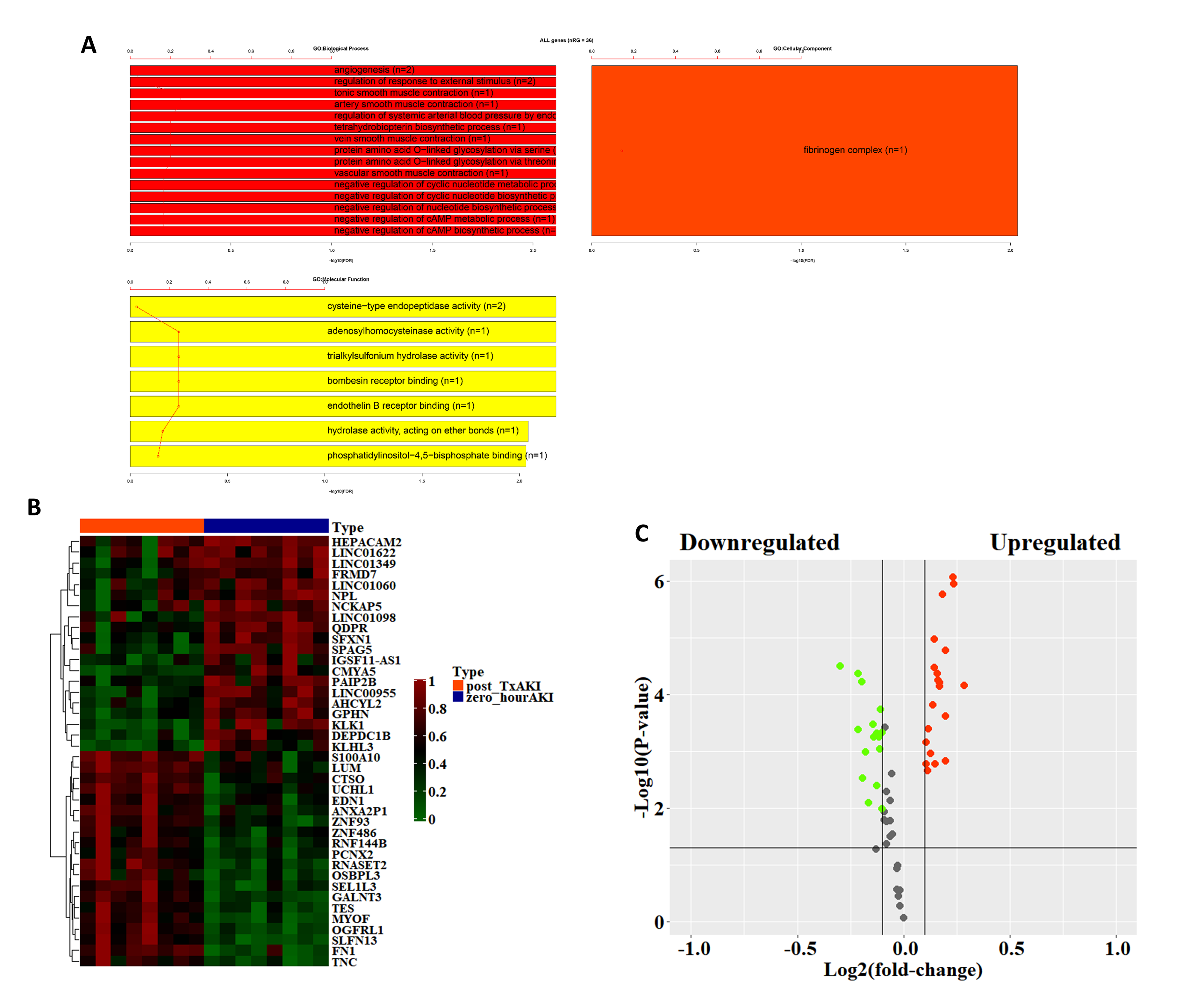

Supplement: Supplementary file 5 — Additional file 5: Figure S5. Detailed information of differentially expressed genes in the tan module. (A) Significantly enriched GO terms/KEGG pathways of differentially expressed tan module genes. (B) Heatmap demonstrating the expression profile of tan module genes. (C) Volcano plot showing the up- or down-regulated tan module genes. [file 40246_2021_363_MOESM5_ESM.tif]

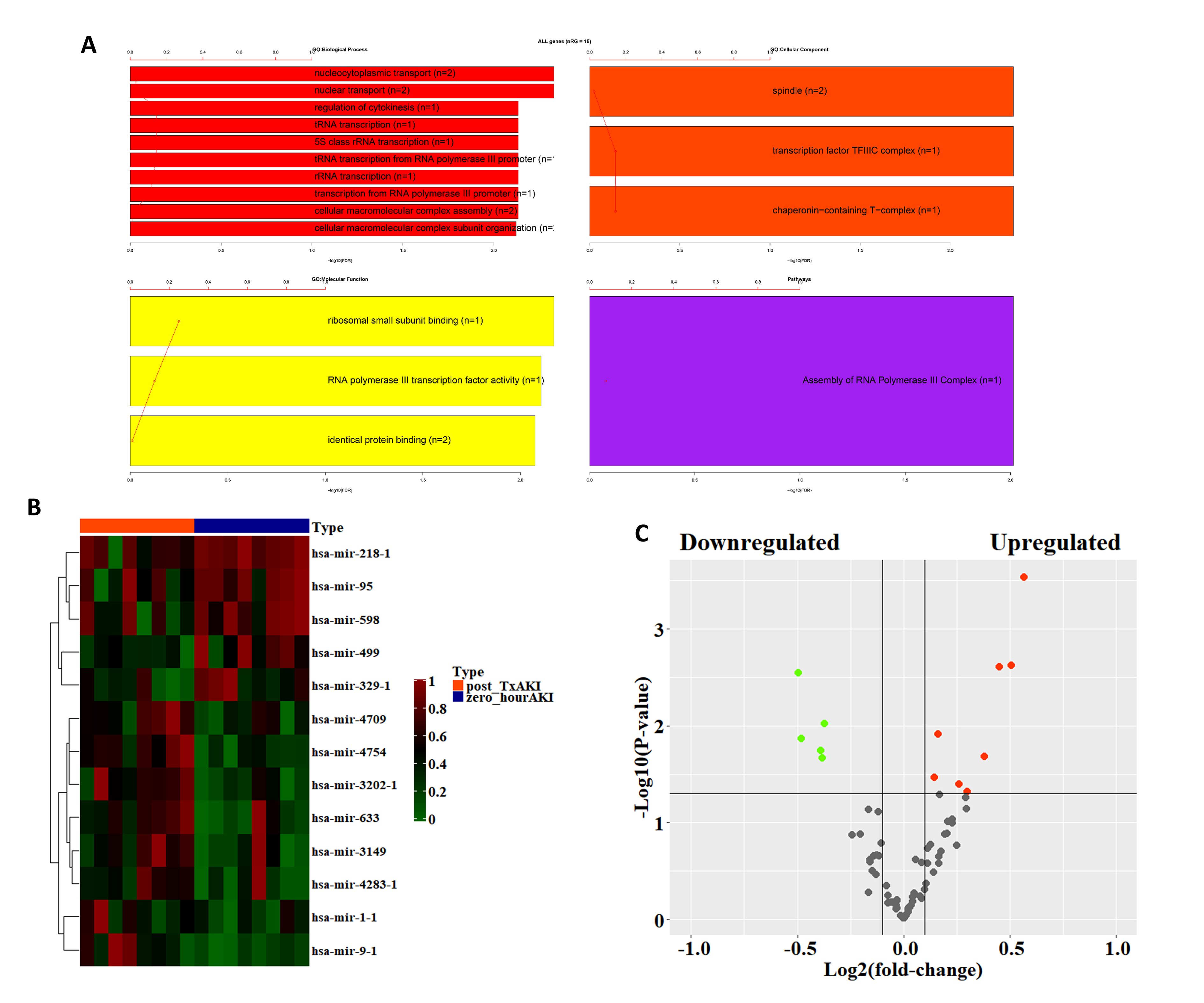

Supplement: Supplementary file 6 — Additional file 6: Figure S6. Detailed information of differentially expressed miRNAs in the blue module. (A) Significantly enriched GO terms/KEGG pathways of the target genes of differentially expressed blue module miRNAs. (B) Heatmap of demonstrating the expression profile of blue module miRNAs. (C) Volcano plot showing the up-/down-regulated blue module miRNAs. [file 40246_2021_363_MOESM6_ESM.tif]

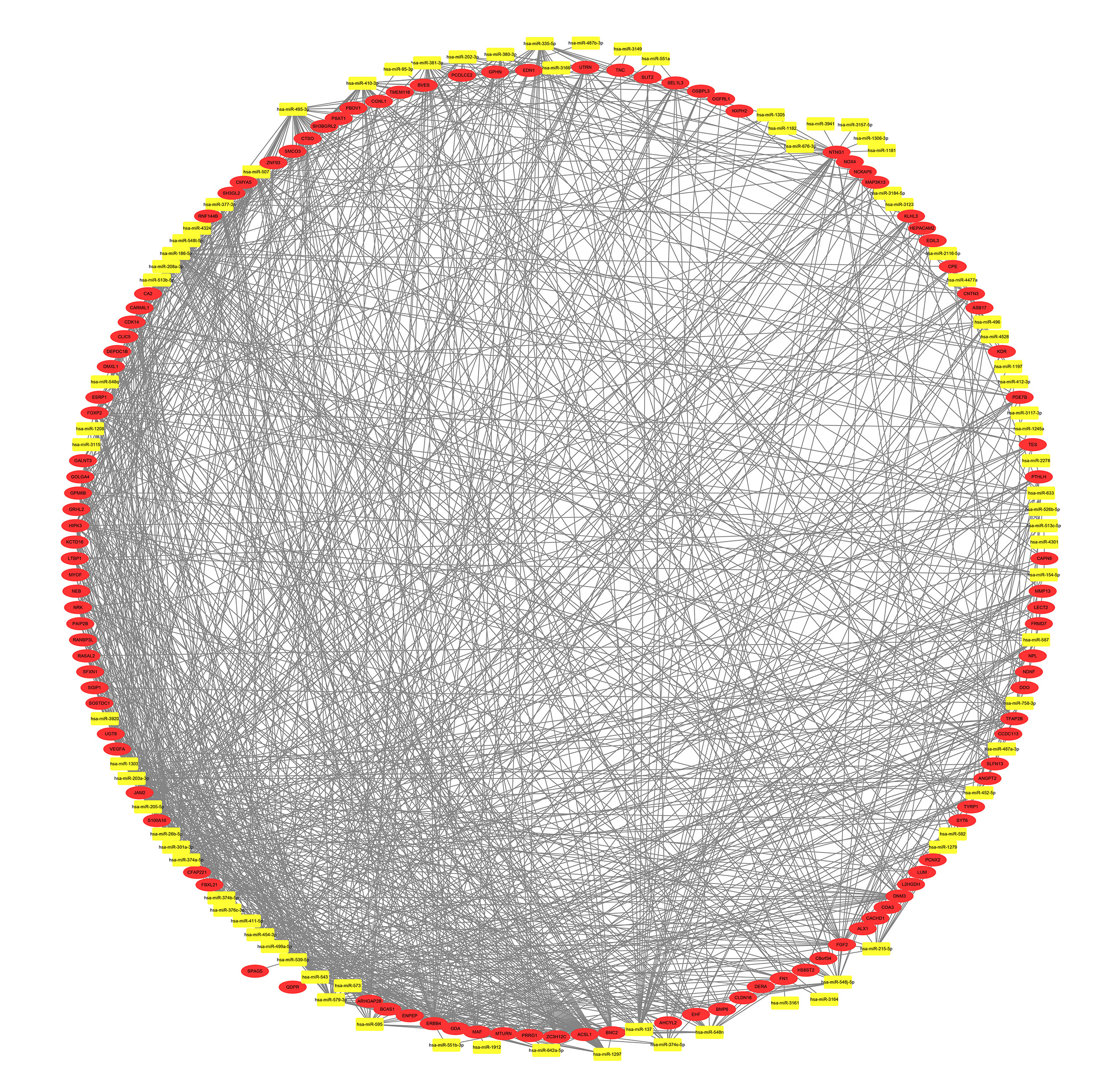

Supplement: Supplementary file 7 — Additional file 7: Figure S7. The preliminary miRNA–mRNA network constructed by interactions of module miRNAs and module mRNAs before setting threshold of confidence. [file 40246_2021_363_MOESM7_ESM.tif]

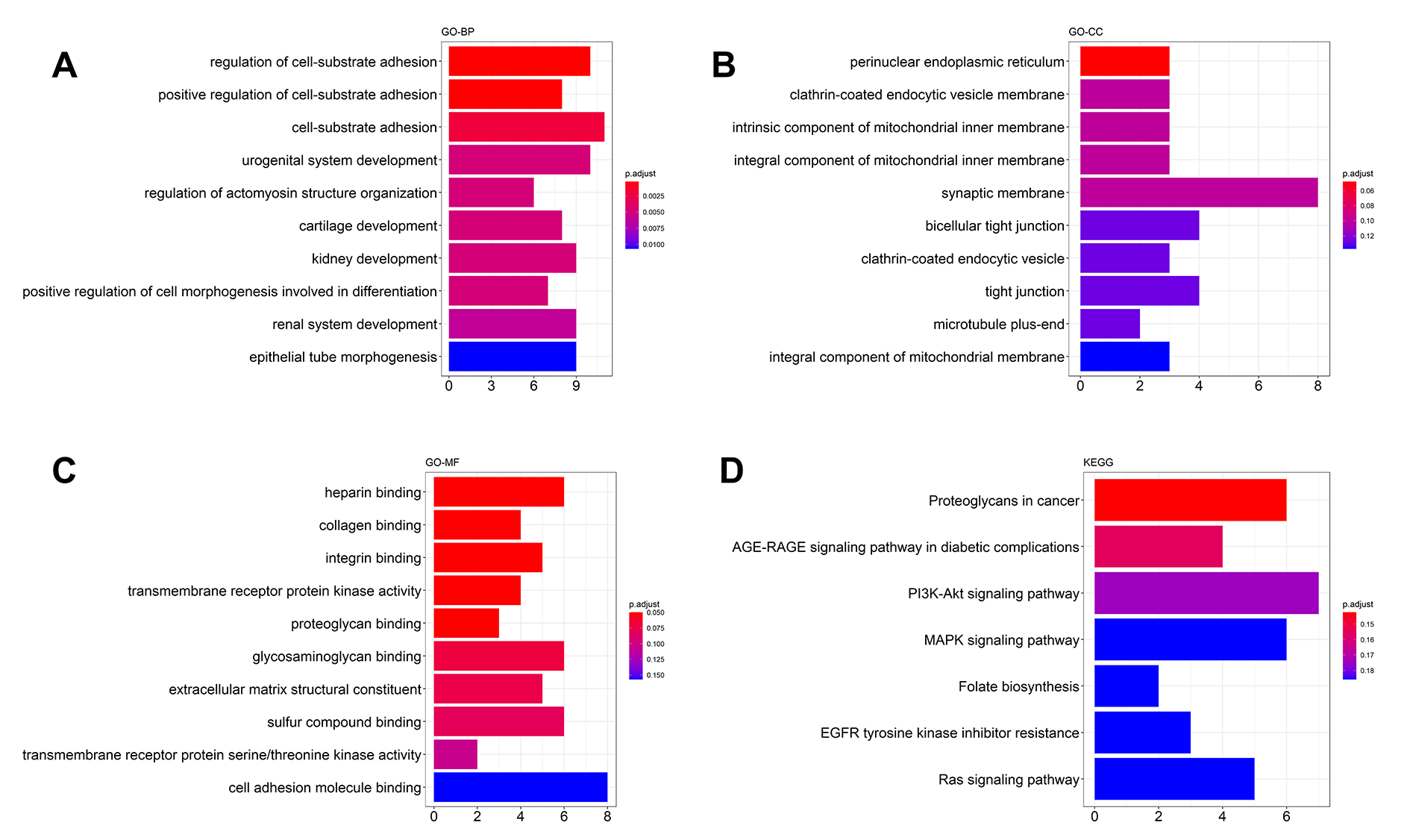

Supplement: Supplementary file 8 — Additional file 8: Figure S8. Functional enrichment analysis of the preliminary miRNA–mRNA network. (A) GO-BP, (B) GO-CC, (C) GO-MF, and (D) KEGG pathway. [file 40246_2021_363_MOESM8_ESM.tif]

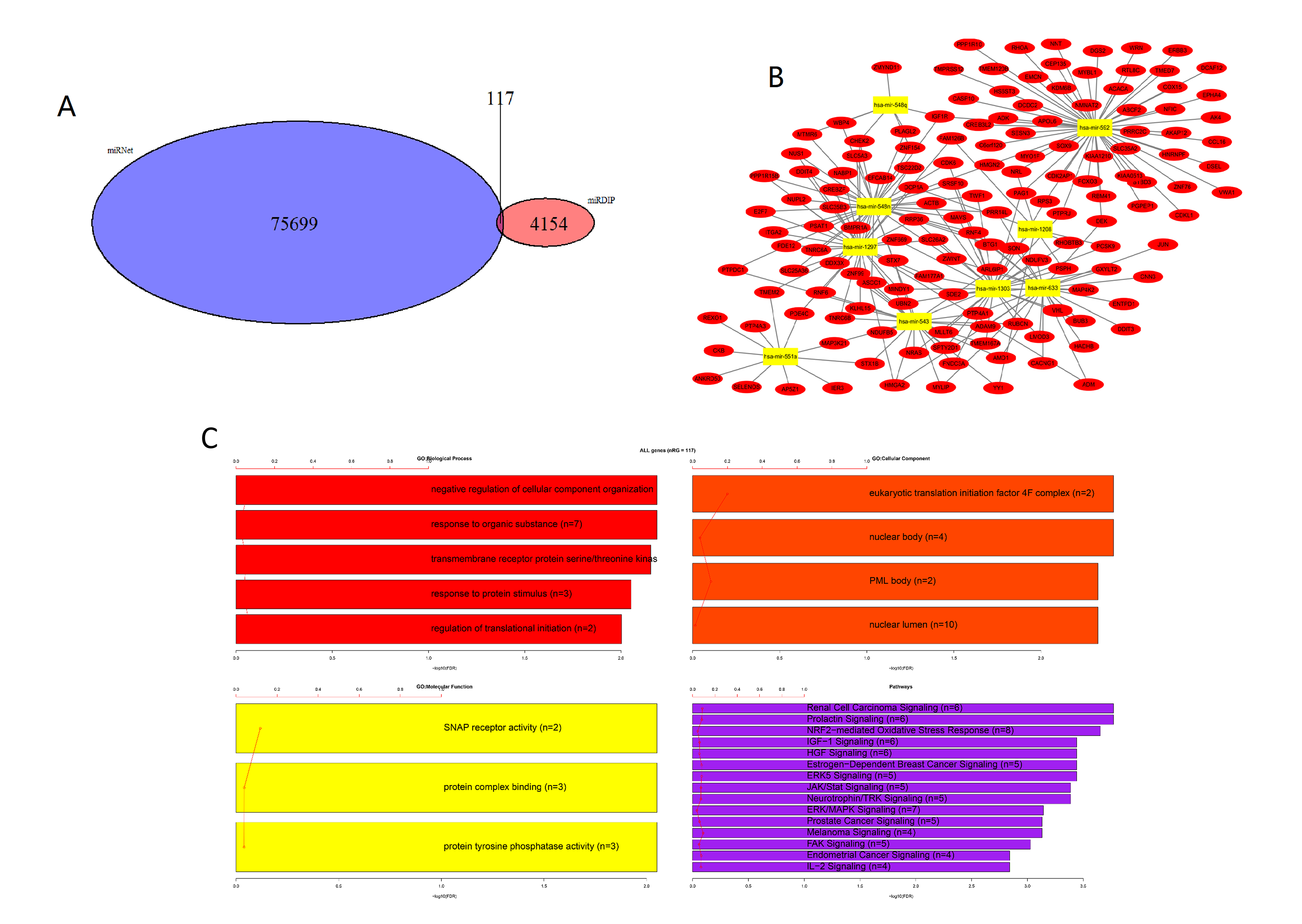

Supplement: Supplementary file 9 — Additional file 9: Figure S9. Cross-validation of the miRNA–mRNA regulatory network. (A) The total number of the identified miRNA–mRNA pairs was 75699 in miRNet database and 4154 in miRDIP database, respectively. A 117 miRNA–mRNA pairs intersection was found and used to construct the (B) miRNA–mRNA regulatory network. The results of the corresponding function enrichment analyses are shown in (C). [file 40246_2021_363_MOESM9_ESM.tif]
